# Skeletal and nonskeletal consequences of hypoparathyroidism

**DOI:** 10.20945/2359-3997000000553

**Published:** 2022-11-10

**Authors:** Barbara C. Silva

**Affiliations:** 1 Centro Universitário de Belo Horizonte Departamento de Medicina Belo Horizonte MG Brasil Departamento de Medicina, Centro Universitário de Belo Horizonte (UniBH), Belo Horizonte, MG, Brasil; 2 Hospital Felício Rocho Unidade de Endocrinologia Belo Horizonte MG Brasil Unidade de Endocrinologia, Hospital Felício Rocho, Belo Horizonte, MG, Brasil; 3 Hospital Santa Casa Unidade de Endocrinologia Belo Horizonte MG Brasil Unidade de Endocrinologia, Hospital Santa Casa, Belo Horizonte, MG, Brasil

**Keywords:** Hypoparathyroidism, fracture risk, quality of life, nephrolithiasis, cataract

## Abstract

Hypoparathyroidism, despite the conventional therapy with calcium and active vitamin D, can lead to skeletal and nonskeletal abnormalities. Chronic hypoparathyroidism is associated with a significant reduction in bone remodeling, increases in areal and volumetric bone density, and improvement in trabecular microarchitecture and in trabecular bone score. Regardless of these advantages in bone mass and microarchitecture, recent data suggest an increased vertebral fracture risk in patients with nonsurgical hypoparathyroidism. Moreover, chronic hypoparathyroidism can lead to abnormalities in multiple organ systems, including the neurological, cardiovascular, renal, neuropsychiatric, ocular, and immune systems. Nephrocalcinosis, nephrolithiasis, and renal insufficiency, as well as decreased quality of life and cataracts, are common in patients with hypoparathyroidism. An increased incidence of hospitalization due to infections and a greater risk of cardiovascular diseases are observed in patients with hypoparathyroidism, particularly in those with nonsurgical disease. All these abnormalities may be because of the disease itself or complications of therapy. We herein reviewed the skeletal and nonskeletal consequences of hypoparathyroidism in patients conventionally managed with calcium and active vitamin D.

## INTRODUCTION

Hypoparathyroidism (HypoPT) is caused by the chronic deficiency or absence of parathyroid hormone (PTH). The diagnosis is confirmed by the finding of hypocalcemia (low serum calcium levels adjusted for albumin or low ionized calcium) accompanied by low or undetectable PTH concentrations and normal magnesium serum levels, on at least two occasions (1–3). HypoPT is considered a rare disease, and its most common cause is an unintentional removal or injury to parathyroid tissue at the time of an anterior neck operation (3–8). Permanent HypoPT has been reported as a complication of 1%-5% of neck surgeries, usually involving the thyroid or parathyroid glands. Other causes include the autoimmune destruction of the parathyroid glands; genetic disorders, such as familial hypocalcemia with hypercalciuria, DiGeorge syndrome, and familial isolated hypoparathyroidism; infiltrative disorders, and ionizing radiation exposure (1,4–6).

Hypocalcemia, the main biochemical consequence of HypoPT, can cause muscle cramps, paresthesia and numbness, life-threatening arrhythmias, laryngospasm, bronchospasm, and seizures. Moreover, PTH deficiency leads to hyperphosphatemia (7,9), which can be associated with ectopic mineralization in soft tissues (9). In bones, chronic deficiency of PTH reduces bone remodeling, with consequent abnormalities in bone density, microarchitecture, and bone strength (1,10–13). The risk of vertebral fractures is increased at least in nonsurgical HypoPT; however, fracture data in patients with postoperative HypoPT, including those at nonvertebral sites, remain controversial (14).

In addition to the abnormalities in bone and mineral metabolism, chronic HypoPT can affect multiple organ systems, including the neurological, cardiovascular, renal, psychiatric, ocular, and immune systems (1,15). These abnormalities may be related to HypoPT itself, complications of the conventional therapy, or both. This narrative review was designed to summarize the skeletal and nonskeletal consequences of HypoPT in patients conventionally managed with calcium and active vitamin D.

### Skeletal consequences of HypoPT

The skeletal consequences of chronic HypoPT conventionally treated with calcium supplements and active vitamin D are described below and summarized in Table 1.

**Table 1:** Summary of the skeletal consequences of hypoparathyroidism

Parameter	Finding	Evidence (reference)
Bone remodeling	Reduced	Bone turnover markers in the lower half of the normal range (12,16). Dynamic histomorphometrical studies of transiliac bone biopsy presenting reduced mineralizing surface and bone formation rate (10–12).
BMD	Mostly increased	BMD by DXA is increased at the LS and hip sites and similar to controls at the radius (5,6,11,17–24). Volumetric BMD at the cortical and trabecular compartments is increased by pQCT (20). Total volumetric BMD is increased by HRpQCT; data on cortical volumetric BMD are diverse (32,33).
Bone microarchitecture	Preserved at the cancellous compartment	TBS is increased or similar to that in controls (29–31). Increased trabecular number and decreased trabecular separation by HRpQCT (32,33). Data on cortical thickness and porosity are diverse according to HRpQCT studies (32,33).
Estimated bone strength	Controversial	Increased (33) or reduced (32) bone strength by finite element analyses of HRpQCT images. Reduced bone strength by microindentation of the tibia (34).

BMD: bone mineral density; DXA: dual-energy X-ray absorptiometry; LS: lumbar spine; pQCT: peripheral quantitative computed tomography; HRpQCT: high-resolution peripheral quantitative computed tomography; TBS: trabecular bone score.

#### Bone remodeling

PTH is required for normal skeletal remodeling, allowing for the replacement of mature bone by newly formed bone. Chronic deficiency of PTH decreases bone remodeling as shown by dynamic histomorphometry and the measurement of bone turnover markers in subjects with HypoPT (10–12). Histomorphometrical studies of transiliac bone biopsies of patients with HypoPT, using tetracycline labeling of bone formation surfaces, revealed unequivocal reductions in remodeling indices, including mineralizing surface and bone formation rate, when compared with healthy controls (1,11,12). Additionally, both bone formation and resorption markers are reduced in HypoPT. The bone formation markers procollagen type 1 amino-terminal propeptide, osteocalcin, and bone-specific alkaline phosphatase are decreased, along with reduced levels of the bone resorption markers serum C-telopeptide and tartrate-resistant acid phosphatase 5b (1). Of note, while histomorphometry of bone biopsies shows a marked reduction in bone remodeling, bone turnover markers are not frankly low but are usually in the lower half of the normal range (12,16), which limits their use in clinical practice to delineate the status of bone turnover in patients with HypoPT.

#### Bone mineral density (BMD) by dual-energy X-ray absorptiometry (DXA)

BMD by DXA is typically increased in patients with HypoPT compared with that in healthy subjects of the same age and sex (5,6,11,17–24). This finding has been demonstrated in different etiologies of HypoPT and in subjects of both sexes on long-term treatment with calcium and vitamin D. BMD is increased at the lumbar spine (LS) and hip sites; however, the highest T- and Z-scores are observed at the LS (5,6,11,17–23). BMD values at the radius are diverse, with some studies showing similarities between HypoPT and controls (17,21–23).

#### Trabecular bone score (TBS)

The TBS is a textural index that evaluates pixel gray-level variations in the LS DXA image, providing an indirect index of bone microarchitecture (25). The TBS predicts fracture risk, independent of BMD and clinical risk factors (26,27). The TBS was evaluated in 52 subjects with HypoPT and compared with that in 27 patients with primary hyperparathyroidism (PHPT). The TBS was normal in patients with HypoPT, in whom the microarchitecture fitted into the classification of low fracture risk, and was significantly greater in patients with PHPT (28). Additional studies have shown that the TBS was either higher in postsurgical (29) and nonsurgical (30) subjects with HypoPT than in controls or similar between these groups (24). The evaluation of 62 patients with postoperative HypoPT also showed a normal mean TBS (1,386) across the entire study population; however, the mean TBS was significantly lower in those with prevalent low-impact fractures (n = 6) than in nonfractured subjects (1,178 *vs.* 1,404; P < 0.001) (31). Similarly, the prevalence of morphometric vertebral fractures increased with declining TBS in a cohort of 152 patients with nonsurgical HypoPT (30).

#### Peripheral quantitative computed tomography (pQCT) and high-resolution pQCT (HRpQCT)

pQCT and HRpQCT have been used to investigate bone quality in HypoPT. Subjects with HypoPT were compared with individuals with PHPT or normal parathyroid function using pQCT of the distal radius (20). The group with HypoPT showed a greater trabecular volumetric BMD (vBMD) and a greater cortical vBMD, cortical area, and thickness than both comparative groups. The total bone area and periosteal and endosteal surfaces were lower in HypoPT than in PHPT.

Using HRpQCT of the radius and tibia, cortical vBMD was higher, and cortical porosity was lower in subjects with HypoPT than in historical controls (32). Cortical thickness and area were decreased in postmenopausal women with HypoPT at both skeletal sites and in women aged < 55 years and men aged < 50 years at the tibia. The trabecular number increased, and trabecular separation decreased, in younger men and premenopausal women with HypoPT. Furthermore, a second HRpQCT study demonstrated a more numerous and less sparse trabeculae in patients with HypoPT than in controls (33). However, the findings on cortical density and porosity were diverse between these two studies, possibly due to differences in the control group and in the methodology used to measure cortical porosity (33).

#### Estimated bone strength

Using HRpQCT and finite element analyses, different results on the estimated bone strength in HypoPT have been described. Cusano and cols. (32) showed either no difference or a decrease in the estimated bone strength between patients with HypoPT and historical controls; however, in a more recent study (33), a much greater failure load was observed in patients with HypoPT than in controls.

The cortical material bone strength was measured using a different approach, namely, microindentation, in 17 patients with HypoPT and compared with that in age-, sex-, and menopausal status-matched controls (34). *In vivo* microindentation assesses the bone material strength index (BMSi), which represents the ability of bones to resist microcrack generation and propagation. The HypoPT group showed lower BMSi than the control group, indicating an abnormal bone matrix in HypoPT, leading to reduced bone strength (34).

#### Fracture risk in HypoPT

As previously noted, HypoPT is associated with increased areal and volumetric BMD and more numerous and less sparse trabeculae. These advantages in bone mass and microarchitecture might contribute to bone strength, which could result in reduced fracture risk in HypoPT. In contrast, patients with chronic PTH deficiency have reduced bone remodeling, which could generate a hyper-mature bone, with compromised capability to repair microdamage and increased risk of fracture. To this end, although observational studies have shown varied results regarding fracture risk in HypoPT (Table 2), it appears that the risk of vertebral fractures is indeed increased in patients with nonsurgical HypoPT (14).

**Table 2:** Fracture risk in patients with nonsurgical and postsurgical hypoparathyroidism

Type of fracture	Etiology of hypoparathyroidism	Fracture risk (reference)
Vertebral	Nonsurgical	Increased (17,30,38).
	Postsurgical	Increased (24,36) or reduced (35).
Clinical fractures	Nonsurgical	Any fracture: similar to the general population (39,40). Fractures in the upper extremities: increased (39).
	Postsurgical	Any fracture: similar to the general population (37,40). Fractures in the upper extremities: reduced (37).

**Postsurgical HypoPT:** The incidence of vertebral fractures was assessed using spinal radiographs in 13 women with postsurgical HypoPT who had undergone total thyroidectomy due to thyroid cancer and compared with that in 20 controls who had also undergone the same surgical procedure but with intact parathyroid function (35). The subjects with HypoPT had a significantly lower incidence of vertebral fractures than patients with normal parathyroid function. In contrast, Mendonça and cols. showed a higher prevalence of morphometric vertebral fractures in 16 patients with HypoPT than in age- and body mass index (BMI)-matched healthy controls (36). More recently, Cipriani and cols. examined the prevalence of vertebral fractures in 50 postmenopausal women with chronic postsurgical HypoPT, compared with 40 age-matched healthy postmenopausal women (24). Vertebral fracture assessment revealed a higher prevalence of vertebral fractures in HypoPT (16%) than in controls (7.5%) (24). The risk of any fracture was also examined in 688 patients with postsurgical HypoPT due to nonmalignant causes and compared with that in 2,064 age- and sex-matched controls (37). No significant difference in the overall fracture risk was observed between the HypoPT and control groups (hazard ratio [HR], 1.03; 95% confidence interval [CI], 0.83-1.29); however, the risk of fractures at the upper extremities was significantly lower in patients with HypoPT (HR: 0.69; 95% CI: 0.49-0.97).

**Nonsurgical HypoPT:** Chawla and cols. evaluated 104 patients with idiopathic HypoPT and 64 controls with normal parathyroid function, with a mean age of ~37 years in both groups (17). A significantly higher prevalence of morphometric vertebral fractures was observed in the HypoPT group (18.3 *vs.* 4.7%), in whom the use of anticonvulsants, as well as its duration, was positively associated with vertebral fractures (17). In another study, 210 patients with nonsurgical HypoPT (62% female; mean age, 39 years) were compared with 2,075 control subjects matched using propensity scores based on age, sex, and comorbid diseases (38). Over a mean follow-up duration of 9.5 years, the incidence of clinical vertebral fractures was higher in patients with nonsurgical HypoPT than in controls (HR: 2.27; 95% CI: 1.09-4.72). Similarly, morphometric vertebral fractures were more frequent in a group of 152 patients with nonsurgical HypoPT (30.9%) than in the control group (7.9%) (30). The risk of any fracture was also investigated in subjects with nonsurgical HypoPT (39). The HypoPT group comprised 180 patients (53% women), with a median age of 49.7 years, and was compared with 540 age- and sex-matched controls. Although the overall fracture risk was similar between the two groups (HR: 1.40; 95% CI: 0.93-2.11), patients with nonsurgical HypoPT had a higher risk of fractures in the upper extremities (HR: 1.93; 95% CI: 1.31-2.85). This finding is in contrast with those of the aforementioned study of postsurgical HypoPT and may be related to the increased risk of seizures and cataracts in patients with nonsurgical HypoPT, resulting in a higher risk of falls and consequent fractures at the upper extremities (39).

**Postsurgical and nonsurgical HypoPT:** In a mixed population of postsurgical and nonsurgical HypoPT, the overall fracture risk was similar between cases and controls, except for subjects with HypoPT related to hypomagnesemia, whose fracture risk was approximately 3.5-fold greater than in controls (40). Finally, a meta-analysis that included data of 1,470 patients with HypoPT, both postsurgical and nonsurgical, and 6,101 control subjects, did not show an increase or decrease in fracture risk of humerus, femur, or any fracture in patients with HypoPT (14). Pooled data showed that the risk of vertebral fractures was 2-fold higher in individuals with HypoPT than in controls (odds ratio [OR]: 2.22; 95% CI: 1.23-4.03); however, in a subgroup analysis, this increased risk was observed only in patients with nonsurgical HypoPT (OR: 2.31; 95% CI: 1.32-4.03) (14). Taken together, these findings show that chronic PTH deficiency can, indeed, reduce bone strength, increasing the risk of vertebral fractures. This is particularly evident in patients with nonsurgical HypoPT, who are more likely to have longer disease duration and to be affected by the disease at an earlier age than subjects with postsurgical HypoPT. Additionally, anticonvulsant therapy in patients with nonsurgical HypoPT may contribute to the increased fracture risk in this group of patients. Data on postsurgical HypoPT and at nonvertebral sites are still controversial.

#### Nonskeletal consequences of HypoPT

The nonskeletal consequences of chronic HypoPT conventionally treated with calcium supplements and active vitamin D are described below and summarized in Table 3.

**Table 3:** Summary of the nonskeletal consequences of hypoparathyroidism

Organ/system	Manifestations
Neuromuscular	Perioral numbness, tingling of the hands or feet, muscle cramping, generalized muscle contractions, bronchospasm, laryngospasm, seizures; the Chvostek and Trousseau signs can be present. Basal ganglia calcifications, which might be associated with symptoms of parkinsonism and other extrapyramidal signs.
Mental health and quality of life	Cognitive impairment, phycological disorders, and reduced quality of life. Increased risk of anxiety, depression, and bipolar affective disorder.
Cardiovascular	Hypotension, bradycardia, impaired cardiac contractility, and arrhythmias. Cardiomyopathy leading to congestive heart failure. Increased risk of cardiovascular disease, ischemic heart disease, cardiac arrhythmias, and stroke.
Kidneys	Hypercalciuria, nephrolithiasis, and nephrocalcinosis. Increased risk of chronic kidney disease.
Eyes	Increased risk for cataracts, and cataract at a younger age than the general population.
Immune system	Increased susceptibility to infections and a greater risk of hospitalization due to infections.

#### Neuromuscular manifestations

Hypocalcemia causes neuromuscular hyperexcitability, generating symptoms that can vary from mild to life-threatening manifestations (7). The degree of hypocalcemia and the rate at which the serum calcium has declined may determine the severity of the symptoms (9). A rapid serum calcium decline in patients with acute PTH reductions following a neck surgery can lead to severe tetany, despite having a serum calcium concentration close to the normal range. Instead, a patient with HypoPT for a prolonged time may be asymptomatic, despite severe hypocalcemia. Other factors, such as concomitant hypomagnesemia and/or alkalosis, can also augment the severity of the symptoms (9).

Neuromuscular symptoms include perioral numbness, tingling of the hands or feet, muscle cramping, generalized muscle contractions, bronchospasm, and laryngospasm, which can lead to respiratory arrest (1,9). Of note, an episode of complete speechlessness, due to spasm of vocalizing muscles, has been associated with severe hypocalcemia (41). The Chvostek and Trousseau signs are physical manifestations of neuromuscular hyperexcitability (42). The Chvostek sign lacks specificity and sensitivity since it is absent in approximately one-third of patients with hypocalcemia and is present in 10% of individuals with normal calcium levels. The Trousseau sign is more sensitive and specific, being present in almost all patients with hypocalcemia and in only 1% of healthy individuals (42).

Seizures can also occur as a neurological manifestation of hypocalcemia in HypoPT (7,9,15,43). They are particularly common in patients with nonsurgical disease (8,39) and can be generalized tonic-clonic or, less frequently, petit mal, partial, or atonic (44). Papilledema, accompanied or not by idiopathic intracranial hypertension, can also be a finding of severe hypocalcemia and improves with the normalization of serum calcium levels (9,45).

The prevalence of basal ganglia calcification (BGC) appears to be greater in individuals with HypoPT than in the general population (5,8,46–48). Although the exact pathogenic mechanisms of BGC in HypoPT require further investigation, an elevated calcium-phosphate product, an increased serum phosphate, and the lack of PTH action on neurons in the basal ganglia may be involved (9,48). A Canadian prospective observational study reported that BGC was present in 15% of patients with surgical HypoPT and in 37% of patients with nonsurgical HypoPT; however, only 40% of the study population underwent brain imaging to look for this complication (47). In a retrospective analysis, BGC was reported in 5 of 14 patients with seizures; however, only 57% of these patients underwent brain imaging (8). The symptoms of parkinsonism and other extrapyramidal signs have been described in patients with HypoPT; however, in some series, these abnormalities improve or completely reverse with treatment for hypocalcemia (9). Although an association of BGC with these neurological manifestations has been proposed (49), the consequences of these brain calcifications remain unclear at this time (48).

#### Cardiovascular complications

Acute hypocalcemia can result in cardiovascular manifestations, including hypotension, bradycardia, impaired cardiac contractility, and arrhythmias (9). Hypocalcemia can increase the ST segment and QT interval, which can progress to torsades de pointes, a life-threatening polymorphic ventricular tachycardia. Hypocalcemia can also lead to electrocardiographic changes, which may mimic a myocardial infarction (9).

In the setting of chronic HypoPT and severe hypocalcemia, several cases of patients with cardiomyopathy leading to congestive heart failure that improves after the correction of hypocalcemia have been reported (50,51). The Danish national registry that evaluated 180 patients with nonsurgical HypoPT showed that the risk of cardiovascular disease (CVD), ischemic heart disease, cardiac arrhythmias, and stroke was approximately 2-fold greater in patients with HypoPT than in controls (39). Two other population-based studies confirmed a greater risk of CVD in patients with nonsurgical HypoPT (38,40). Of note, an analysis involving 431 patients with HypoPT of diverse etiologies showed a greater risk of CVD in those with longer duration of HypoPT, with a 3.7-fold increased risk in those who had been suffering from HypoPT for >20 years compared with that in those with a disease duration of <7 years (52). The risk was also greater in patients with lower serum calcium concentrations and in those with ≥ 4 episodes of hypercalcemia during follow-up (52).

In contrast to these data, the risk of cardiac arrhythmias and any CVD was similar between cases and controls in a large Danish national study, which evaluated 688 patients with postsurgical HypoPT and 2,064 matched controls (37). Similarly, a Scottish population-based study found no increased risk of CVD in patients with postsurgical HypoPT compared with that in controls (40).

#### Renal complications

Conventional therapy of HypoPT with calcium supplements and active vitamin D may result in hypercalciuria, which is a risk factor for nephrolithiasis and nephrocalcinosis (53). An increased risk of chronic kidney disease (CKD) (estimated glomerular filtration rate [eGFR] < 60 mL/min/1.73 m^2^) has also been described in patients with HypoPT on conventional therapy (5,8,37,39,40,43,52–55). A recent systematic review reported higher rates of nephrolithiasis (up to 36%) and CKD (up to 41%) in chronic HypoPT than in the general population (53). Similarly, population-based studies of HypoPT compared with matched controls from the background population showed that patients with HypoPT, both surgical and nonsurgical, have a 3-10-fold higher risk of CKD and a 2-4-fold greater risk of kidney calcifications (37–40). A large retrospective study, using a managed care claims database in the United States (8,097 patients with HypoPT and 40,485 controls), confirmed these observations (56). In models adjusted for baseline characteristics, patients with HypoPT had an increased risk of developing CKD (HR: 2.91; 95% CI: 2.61-3.25), CKD stage progression (HR: 1.58; 95% CI: 1.23-2.01), and progression to end-stage kidney disease (HR: 2.14; 95% CI: 1.51-3.04) (56). The risk of CKD among patients with HypoPT appears greater in those with longer duration of HypoPT, a higher median calcium-phosphate product, and a higher frequency of episodes of hypercalcemia (52).

#### Psychiatric complications and quality of life (QoL)

Numerous studies have shown that patients with HypoPT on conventional therapy have a higher prevalence of cognitive impairment, phycological disorders, and/or reduced QoL than a control group or the general population (37–40,54,57–61). An increased risk of anxiety, depression, and bipolar affective disorder has been reported in patients with postsurgical and nonsurgical HypoPT (37,38,40,59,60). Using the 36-Item Short-Form Health Survey (SF-36), a validated nondisease-specific instrument for the overall assessment of health and well-being, several studies have shown impairments in physical and mental health domains in individuals with HypoPT (54,58,59,62). Recently, a disease-specific measure to capture the impact of HypoPT on functioning and well-being has been developed and validated: the Hypoparathyroidism Patient Experience Scale-Impact (HPES-Impact) (63,64). These studies evaluated 42 patients with HypoPT from the United States, including 35 (83%) women and 36 (86%) with postsurgical HypoPT, with a mean age of 53 years and a median disease duration of 9 years. The results showed substantial impacts on physical functioning, including the ability to exercise; daily life, including the ability to perform things around the home; psychological well-being, including feeling anxious and frustrated; and social well-being, including the ability to participate in social activities and relationships (63). Additionally, symptoms, such as tingling/numbness/paresthesia, muscle cramping, and physical fatigue, and cognitive dysfunction, including impaired memory, impaired ability to have a conversation, and lack of concentration, have also been reported (64).

#### Cataracts

An association between cataracts and HypoPT has been described for over 100 years (65). This finding was confirmed by population-based studies that showed an approximately 2-4-fold greater risk of cataracts in individuals with nonsurgical HypoPT (38,39) and a 1.8-fold higher risk of cataracts in patients with postoperative HypoPT (40) than in matched controls. In addition to the increased risk of cataracts, patients with nonsurgical HypoPT were diagnosed with cataract at a younger age than the general population (53 *vs.* 60 years) (39). Similarly, in a study involving 27 patients with idiopathic HypoPT compared to 25 controls, patients with HypoPT underwent cataract surgery at a younger age than controls (34 ± 16 vs. 58 ± 11 years; P < 0.001) (65). In contrast, in the Danish national registry of postoperative HypoPT, no differences in the incidence of cataracts and the age of onset were observed between cases and controls (37).

An observational study found cataracts in 11 of 20 women with postsurgical HypoPT who were screened for the disease (66). Patients with cataract tended to be older and have a longer duration of HypoPT (66). Of note, a higher frequency of cortical cataracts was found in this study; however, typical age-related cataracts are more likely to be nuclear (15). In another cohort of individuals with HypoPT, the prevalence of cataracts by self-report was only 2.7% and 10% in 292 patients with surgical HypoPT and 30 patients with nonsurgical HypoPT, respectively (8). These data suggest that cataracts are underdiagnosed in patients not actively screened for the disease.

#### Infections

Population-based studies have found that patients with HypoPT have an increased susceptibility to infections. The studies by Underbjerg and cols. showed a greater risk of hospitalization due to infection in postsurgical (HR: 1.42; 95% CI: 1.20-1.67) and nonsurgical HypoPT (HR: 1.94; 95% CI: 1.55-2.44) than in the general population (37,39). A greater incidence of inpatient admission due to infection was also observed in a mixed population of nonsurgical and surgical HypoPT (40). The risk factors for infections appear to be increased HypoPT duration, hyperphosphatemia, and higher frequency of hypercalcemia episodes (52).

Although the cause for this increased susceptibility to infections in HypoPT is unknown, hypocalcemia and low PTH levels may be involved (67). Calcium signaling plays an important role in the immune system, such as mast cell degranulation, target cell lysis by cytotoxic T cells, and lymphocyte differentiation (68). Immune cells express PTH receptor 1 on their surface. In a recent study involving 20 patients with postsurgical HypoPT and 20 age- and sex-matched controls, PTH receptor 1 was expressed in all lineages of peripheral blood mononuclear cells; however, the percentage of cells expressing PTH receptor 1 was lower in HypoPT than in controls (67). Additionally, patients with HypoPT had a lower count of monocytes, total, regulatory, and naive CD4+ T lymphocytes and a higher CD3-CD56+ natural killer cell count than controls. Serum calcium and PTH levels were directly correlated with monocyte and lymphocyte counts and inversely correlated with CD3-CD56+ natural killer cells (67). Further studies are needed to clarify whether these abnormalities contribute to the increased risk of infectious diseases in patients with HypoPT.

### Summary

HypoPT is associated with skeletal and nonskeletal abnormalities, despite the conventional therapy with calcium and active vitamin D. In the skeleton, chronic PTH deficiency reduces bone remodeling and increases areal and volumetric BMD. Using HRpQCT, trabecular number is greater, and trabecular separation is lower, consistent with the finding of a normal TBS in HypoPT. Despite these obvious advantages in bone density and microarchitecture, vertebral fracture risk is increased in nonsurgical HypoPT. Fracture data in patients with postoperative HypoPT, including fractures at nonvertebral sites, remain controversial.

HypoPT leads to hypocalcemia, which causes neuromuscular hyperexcitability and cardiovascular manifestations, such as hypotension and arrhythmias. An increased risk of CVD has been observed in chronic HypoPT, particularly in patients with nonsurgical HypoPT. Nephrolithiasis, nephrocalcinosis, and CKD are also more prevalent in HypoPT than in the general population. Patients with HypoPT on conventional therapy also have a higher risk of infections, cataracts, cognitive impairment, phycological disorders, and reduced QoL.

These abnormalities may be related to HypoPT itself, may be caused by the complications of conventional therapy, or both. Further research is needed to determine whether the treatment with PTH or other interventions can decrease the risk of these complications in individuals with HypoPT.
